# Mechanisms of Proteolytic Enzymes and Their Inhibition in QM/MM Studies

**DOI:** 10.3390/ijms22063232

**Published:** 2021-03-22

**Authors:** Brigitta Elsässer, Peter Goettig

**Affiliations:** Structural Biology Group, Department of Biosciences, University of Salzburg, Billrothstrasse 11, 5020 Salzburg, Austria; brigitta.elsaesser@sbg.ac.at

**Keywords:** proteases, enzymes, qm/mm, quantum chemistry

## Abstract

Experimental evidence for enzymatic mechanisms is often scarce, and in many cases inadvertently biased by the employed methods. Thus, apparently contradictory model mechanisms can result in decade long discussions about the correct interpretation of data and the true theory behind it. However, often such opposing views turn out to be special cases of a more comprehensive and superior concept. Molecular dynamics (MD) and the more advanced molecular mechanical and quantum mechanical approach (QM/MM) provide a relatively consistent framework to treat enzymatic mechanisms, in particular, the activity of proteolytic enzymes. In line with this, computational chemistry based on experimental structures came up with studies on all major protease classes in recent years; examples of aspartic, metallo-, cysteine, serine, and threonine protease mechanisms are well founded on corresponding standards. In addition, experimental evidence from enzyme kinetics, structural research, and various other methods supports the described calculated mechanisms. One step beyond is the application of this information to the design of new and powerful inhibitors of disease-related enzymes, such as the HIV protease. In this overview, a few examples demonstrate the high potential of the QM/MM approach for sophisticated pharmaceutical compound design and supporting functions in the analysis of biomolecular structures.

## 1. Introduction

Traditionally, enzymatic mechanisms are the domain of experimental and empirical research, however, many details on the molecular level are elusive, in particular, when the time scale of the reactions is very small. This assertion holds true for the mechanisms of proteolytic enzymes, which were discovered and investigated already in the 1800s. Early observations related to their mechanisms date back to around 1900, as pointed out in a recent review with a historical perspective by Judith Bond [1]. Among the proteolytic enzymes, pepsin may serve as a paragon for protease research, with all its diverse aspects. Porcine pepsin isolated from stomach was (besides trypsin) the first protein cleaving enzyme discovered and termed a “proteose”, which was later replaced by “protease” [2]. Remarkably, pepsin was capable to both degrade and synthesize proteins in accordance with its catalysts nature, similar to trypsin [3]. Pepsin was the first protein, which formed crystals that exhibited a distinct diffraction pattern [4,5]. Nevertheless, systematic enzymological studies of pepsin began in the 1960s, revealing mechanistic details on the molecular level, as summarized by Fruton [6]. Not surprisingly, and following bovine α-chymotrypsin, the second protease structure which was determined at atomic resolution, was the one of porcine pepsin [7,8].

Meanwhile, a large amount of experimental data and commonly accepted theories of mechanistic models has accumulated. Since the corresponding literature is so vast, the interested reader should have a look at the MEROPS data base of proteolytic enzymes and their inhibitors, which contains comprehensive information and most of the links to the relevant and recent literature [9]. The major peptidase families are aspartic (A), cysteine (C), metallo (M), serine (S) and threonine (T). In addition, families exist with only a few and less significant members: glutamic (G), which are related to aspartic, mixed (P), asparagine (N) and unknown (U). Furthermore, the corresponding sections of the *Handbook of Proteolytic Enzymes* include concise chapters, with details on the respective proteolytic mechanisms. Their most important elements are: 1. a nucleophile, such as Ser, Thr, or Cys that attacks the carbonyl C atom of the scissile bond; 2. a catalytic H_2_O, which can be the primary nucleophile as in metallo-proteinases, whereas it may be a secondary nucleophile or a proton donor; 3. a general acid and base system, which transfers a proton to the new N-terminal amino group; 4. an oxyanion hole or equivalents, which stabilize the carbonyl O in the tetrahedral intermediate of the substrate.

Most acidic proteases or peptidases require a low pH for efficient activity and two Asp residues to activate the catalytic water molecule, but rare variants from fungi are known, in which a Glu and a Gln efficiently replace the aspartates [10]. In metallopeptidases, usually two His residues coordinate the catalytic Zn^2+^, while His, Glu or Asp can serve as third ligand [11]. The fourth ligand of the metal ion is always the catalytic water, in many cases positioned close to an activating Glu side chain. If co-catalytic Zn^2+^ ions are present, the coordination pattern shows more variation with respect to ligands. Cysteine peptidase and serine peptidase mechanisms are similar, but as the Sγ atom is more nucleophilic and more prone to oxidation than the Oγ, the activity of cysteine peptidases is restricted to a reducing environment, such as the cytosol [12]. Overall, catalytic triads of cysteine peptidase exhibit variations, with Glu, Asn, His as third residue or none at all in dyads, while the oxyanion hole is more diverse as well. Serine and threonine peptidases depend on the activation of their γ-OH, whose nucleophilicity has to be enhanced in a classical catalytic triad or by the N-terminal α-amino group [13]. Some serine proteases differ with Ser-His-His or Ser-Asp-Glu triads or Ser-His and Ser-Lys dyads. Typically, the role of the His as a general base and proton acceptor is crucial for the formation of the tetrahedral intermediate in serine proteases, followed by general acid catalysis of the protonated His, resulting in the formation of the acyl intermediate and release of the amine or P1′ product. The hydrolysis of the acyl intermediate continues with His acting as general base and acid and ends with release of the carboxyl or P1 product. Similarly, in threonine peptidases, e.g., in active proteasome subunits, tetrahedral, and acyl intermediate form the γ-OH of Thr1, whereas its α-amino group acts as general base and acid, as well as oxyanion hole equivalent [14]. Besides the five clearly defined and well-studied peptidase classes, unusual peptidases in eight families of the “unknown” type (U) exist with yet uncharacterized active sites and mechanisms [15].

The hybrid QM/MM method is an excellent tool to model the reaction mechanisms of peptidases, whereby the whole enzyme-substrate-solvent complex is simulated. While the active site, where bond cleavage and formation occur, is studied by a quantum chemical method, the rest of the enzyme and the surrounding solvent are described by the method of molecular mechanics. By contrast, force field based molecular dynamic approaches cannot handle chemical bonds forming or breaking. In addition, QM methods allow to describe metal coordination spheres more accurately than classical molecular dynamics, which is especially relevant for the metalloproteases. 

Interestingly, numerous QM/MM studies focus on aspartic proteases, namely HIV protease and human β-secretase (BACE), which are both related to pepsin. Most likely, the reasons for this preference are their relatively simple mechanism, which requires less computing power, and their roles in AIDS and Alzheimer’s, respectively. Thus, QM/MM approaches will contribute to understand the molecular mechanisms of pharmaceutical targets and help to develop new drugs for medical applications. Unfortunately, the specialized publications in this area of research contain many computational and biophysical details, which makes them hardly accessible for experimentalists working on the same enzyme-substrate systems. This overview aims to demonstrate that it is worthwhile to study papers on QM/MM derived reaction mechanisms, which often agree very well with experimental data.

## 2. Principles of Quantum Mechanical Molecular Mechanics Calculations for Proteins

**Figure 1:**
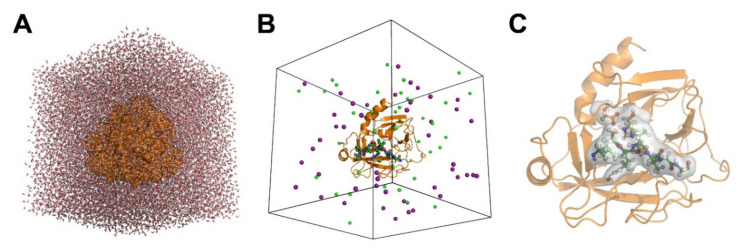
Visualization of the setup for a QM/MM simulation. (**A**) The molecule of interest, a protease- substrate complex, is centered in a water filled cubic box. Both molecules are depicted with their surface in orange. (**B**) The MD or MM simulation box shows the protease in ribbon representation (orange), the substrate as ball-and-stick model (green), and the counterions Na^+^ (purple) and Cl^−^ as spheres. (**C**) Most atoms of the protease in ribbon representation were omitted for clarity, while the QM region with the substrate and the catalytic residues, displayed as ball-and-sticks, is defined by a surrounding, transparent surface. The MM–QM interface requires a special treatment, e.g., by using link atoms.

Although most researches in the field follow several standard procedures, many of them employ different levels of theory, exchange correlation, and various basis sets depending on the respective software and available computing power. A comprehensive review, which demonstrates the common features of the most relevant QM/MM approaches was written by van der Kamp and Mulholland [16]. Usually, protein coordinates from the Protein Data Bank (PDB) (http://www.wwpdb.org/ 3 March 2021) serve as basis for the QM/MM calculations, preferably with a good resolution, i.e., around 2 Å or better [17]. A bound substrate analog or inhibitor is a desirable feature of the experimental coordinates. The protein substrate system is equilibrated after addition of hydrogen atoms and centered in a water-filled periodic box or sphere with counter ions, such as Na^+^ and Cl^−^, in order to maintain overall charge neutrality at a given pH [18]. Protonation is calculated according to calculated pK_a_ values for enzyme and substrate residues. Usually, the enzyme-substrate complex or more general, the solute, is placed in the box or sphere in a way that all of its atoms are at least 8 Å away from the boundaries. Consequently, it may contain tens of thousands of water molecules and way more than overall 100,000 atoms, including hydrogen atoms. About 50 to 100 atoms of the active site, including the substrate or its analog, are treated as QM region, while the rest of the enzyme and the surrounding solvent comprise the MM region (Figure 1).

The two ways of calculating the total energy of the system are the additive and the subtractive method, with either E_total_ = E_QM1_ + E_MM2-1_ or E_total_ = E_QM1_ + E_MM12_ – E_MM1,_ [19]. In the latter case, a MM calculation for both the QM region 1 and the MM region 2 is performed and the MM term for region 1 subtracted. The MM–QM boundary is usually capped by hydrogen atoms and is treated by special algorithms. In order to reduce the degrees of freedom in the MM–QM boundary, link atoms are defined and corresponding constraints set, which handle the bonds and orbitals at the hybrid interface [20,21]. 

The first step of each reaction mechanism calculation is to equilibrate the entire solvent-enzyme-substrate structure in a series of MD annealing runs at temperatures 50, 150, 200, 250 and 298.15 K [18]. The MD calculations can proceed in several alternating steps, such as starting with fixed positions of the atoms in the QM region and afterwards the atomic positions in the MM region are fixed, while the atom positions in the QM region are optimized at the given level of theory and wave functions. For example, in the often used *ab initio* approach of Car-Parinello MD, the wave function is computed at the beginning of the simulation according to the Schrödinger equation. Then, the resulting equilibration stage of the structure should undergo multi-region optimization. This method performs a sequence of alternating optimization cycles of the QM and MM regions. During the MM region optimization the electrostatic field in the QM region is usually represented by a set of effective charges, which greatly increases the efficiency. The effective charges are recalculated in each optimization cycle by fitting the electrostatic field outside the QM region to that produced by the full electron density representation. Several optimization cycles are carried out until convergence is reached. A particular problem of QM/MM computation was the treatment of the electrostatic contribution of the MM region, which used to be added with partial charges in the QM region and was termed mechanical embedding [22]. Ligand or substrate charges in the MM and complete QM/MM simulation may use a restrained electrostatic potential (RESP). More sophisticated approaches use electrostatic embedding, in which the electrostatics of the MM region influences the wave function of the QM region. In polarized embedding, the QM region retroacts on the MM region, like in a mutual feedback situation. Among the various QM models are semi-empirical ones, coupled cluster (CC) methods, time-consuming density functional theory (DFT) computation with B3LYP as exchange correlation and *ab initio* calculations for molecule orbitals, such as the second-order Møller–Plesset method (MP2) [23,24]. In order to treat the MM region and its interaction with the QM region, an appropriate force field is applied, e.g., AMBER99SB-ildn, AMBER14, or CHARMM27. The calculation of the reaction itself benefits from information on the transition states, as derived from corresponding transition state analog or inhibitor structure coordinates. Ideally, a computed minimum energy pathway (MEP) along the reaction coordinate results in a free energy profile with minima representing reaction intermediates (INT1, INT2, …) and maxima or barriers representing transition states (TS1, TS2, …). Nevertheless, relative potential energies can be calculated, as well. In addition, multiple starting conformations can be explored, which may reveal alternative MEPs. An informative overview of the most relevant aspects of QM/MM simulation for reaction mechanisms was written by Hu and Yang [25]. Starting from a stationary point such as reactant, enzyme substrate complex or product state, a sequence of constrained optimizations are performed to study assumed proton transfers, or bond cleavage and formation. Herein, one or two harmonic constraints between the participating atoms (only in strength and not for the direction) are imposed to drive the system over the intermediate, transition states and reaction barriers to the product state while at the same time the MM system, which was initially equilibrated to the reactant structure, is allowed to adjust to these changes. When a reasonable estimate of an intermediate is obtained, the constraints are lifted, and a sequence of optimization and dynamical relaxation steps is applied to the system, similar to those discussed above. Consecutively, the trial reaction is optimized between two fixed points in several steps and the free energy of the coordinate along the reaction pathway is calculated using, e.g., free energy perturbation, umbrella sampling, or adaptive biasing force. The time scale of QM/MM simulations is in the range of 100 ps to 1 ns and, thus, requires additional sampling methods, such as metadynamics, accelerated MD, or transition path sampling. In case the reaction could take alternate routes, e.g., by two different ways to protonate the amide nitrogen of a hydrolyzed peptide bond, be it direct or water mediated, they all should be simulated. According to transition state theory (TST) the highest energy barrier of an overall enzymatic reaction can be compared to the free energy obtained from experimental kinetic parameters. Based on the Gibbs-Helmholtz, Van ‘t Hoff and Arrhenius equations ∆G = ∆H – T∆S, ∆G = –RT ln K_eq_, k(T) = A exp(–ΔG^‡^/k_B_T) the Eyring equation allows to derive the free activation energy ∆G^‡^ of a reaction from k_cat,_ Equation (1), which is the turnover number of an enzyme [26]:

(1) $$k_{\mathrm{cat}}=\frac{k_{B}T}{h}•\;e^{-\Delta G^{‡}/RT}$$

Hereby, k_B_, h and R are the Boltzmann, Planck, and universal gas constants. Several publications discuss experimental free energies in light of the corresponding data from QM/MM results, which often agree very well. Usually, ΔG^‡^ is calculated relative to the Michaelis complex, whose formation is often not explicitly mentioned as the first step of the overall reaction mechanism.

A recent comparative study on the five major protease classes found that Cys proteases require the lowest free activation energy, whereas the one of aspartic proteases is nearly three times higher, when the dipeptide H_2_N-Gly-Gly-CO_2_H is cleaved (Table 1) [27].

## 3. Mechanisms of Aspartic Proteases

**Figure 2:**
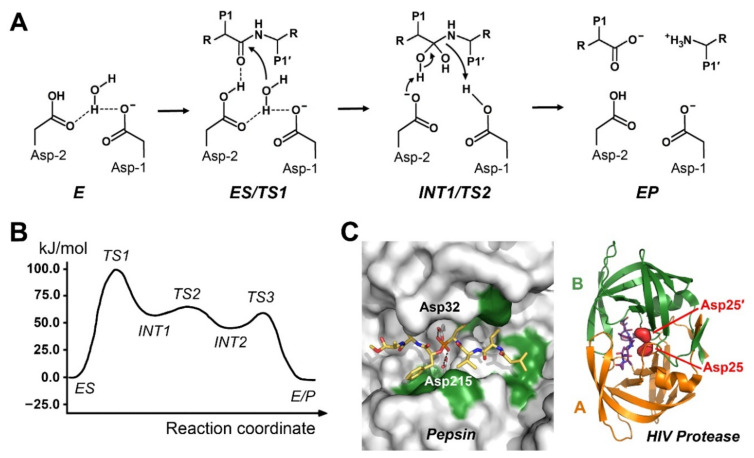
Mechanism of aspartic proteases. (**A**) Essentially three major steps takes place, including formation of the Michaelis complex 1. Nucleophilic attack by an activated water molecule with transition state 1 (TS1) and formation of a tetrahedral intermediate (INT). 2. Nitrogen conversion (TS2). 3. Fission of the scissile bond and release of the products with new C- and N-termini (EP). Residue numbering corresponds to pepsin. Two relevant aspartic proteases are shown in Figure 2C. (**B**) Free energy profile for the pepsin-like protease renin, with an additional reaction step including INT2 and TS3, according to Bras et al. (2012) [33]. (**C**) Left panel: Active site of pepsin with a phosphonate inhibitor, mimicking TS2 (PDB 1QRP). White areas are polar, green areas are hydrophobic. Right panel: The dimeric HIV protease has one catalytic Asp25 (red spheres) per monomer (PDB 4HVP). A peptidic inhibitor (purple sticks) is bound to the active site as ES analog.

As described above, the most relevant aspartic proteases are pepsin-like or related to them. An early comprehensive overview on structures and mechanisms of the pepsin family proteases, among them the cathepsins D and E, as well as parasite and plant pepsins, was written by Ben Dunn [28]. The pepsin-like proteases are monomeric and comprise specificity pockets from S5 to S3′, while the retroviral versions, the retropepsins, are active as dimers, with distinct specificity pockets from S3 to S3′. Usually, both catalytic aspartate residues, 32 and 215 in pepsin, are located in D-T/S-G-T/S motifs [10]. The pH optimum of human pepsin I activity lies around 2.5, corresponding to its secretion into stomach, whereas human BACE1 (β-secretase) and HIV protease display optimal activity around pH 5.0 and 6.0, respectively [29,30,31]. Structural data show that both carboxylates and a catalytic water molecule are coplanar, whereby the carboxylate group of Asp215 carries a negative charge in contrast to the neutral one of Asp32, which are both stabilized by the neighboring hydroxyl groups of Ser35 and Thr218 [32]. The generally accepted mechanism after formation of the enzyme-substrate or Michaelis complex starts with a nucleophilic attack of the activated water molecule on the carbonyl C atom of the scissile bond, including formation of a tetrahedral *gem*-diol intermediate and a proton transfer to the carbonyl O atom. The second step includes the so-called nitrogen inversion, a concerted rearrangement of electron pairs and proton transfer to the amide NH, resulting in cleavage of the peptide bond and product release (Figure 2).

To date, no QM/MM study is available, which describes the overall peptide cleavage mechanism of pepsin. According to a QM simulation for the active site of pepsin both hydrogens of the catalytic water bind the negatively charged carboxylate of Asp32, whereas the oxygen forms a hydrogen bond to the Oδ1 of Asp215 [34]. One of the water H bridges the Asp32 Oδ1 and the Asp215 Oδ2, contrary to the conformation depicted in Figure 2. Pepsin-like human BACE1 (β-secretase) is associated with neurons and their myelinization, while it processes the amyloid precursor protein, which plays an important role in Alzheimer´s disease. QM/MM calculations for the nucleophilic attack catalyzed by BACE-1 were included in a comparative paper on various aspartic proteases [35]. Using the ONIOM method with mechanical embedding, the cleavage by BACE1 was computed for the wild type bond Met2-Asp3 and the disease-promoting Swedish variant Leu2-Asp3 [36]. It turned out that calculated QM/MM free energy barriers, e.g., 72.0 kJ/mol for the Swedish variant agreed very well with the experimental values (75.4 kJ/mol). A systematic QM/MM X-ray refinement study for eight possible protonation states of both catalytic Asp residues in BACE1 strongly favored protonation of the inner oxygen of Asp32. The question of the initial protonation state of either Asp32 or Asp215 has not an unambiguous answer, in particular, as different experiments confirm that both states are possible for the same pepsin-like protease, such as plasmepsins [37]. Based on crystal structure data, QM/MM calculations of plasmepsin IV bound to a tetrahedral transition state mimicking inhibitor, favored protonation of Asp214, in contrast to experimental data for pepsin [38]. Plasmepsins are expressed by the parasite *Plasmodium falciparum* and represent highly interesting targets for anti-malaria drugs. A study with plasmepsin II and a similar inhibitor equally showed a preference for a protonated Asp214 [39]. The free binding energy terms ΔG_Elect_−QM/MM of the two different protonation states with bound inhibitors showed differences of 11.1 and 15.6 kJ/mol, respectively. Cathepsin D is a monomeric intracellular aspartic protease from lysosomes and involved in various diseases, such as breast cancer, which makes it an interesting target for inhibitor interaction analyses by QM/MM using pepstatin A derivatives [40]. Similarly, five steps were assumed for mouse renin, a monomeric, blood pressure regulating aspartic protease, although the calculated and experimental free activation barriers agreed only moderately [33].

In dimeric retropepsins, such as human T-cell leukemia virus type 1 (HTLV-1) protease, Asp32 and its counterpart Asp32’ in the second molecule share one proton in the substrate bound state, whereas both catalytic Asp residues are unprotonated in the ligand-free form [41]. A combined neutron diffraction and QM/MM study corroborated a proton shared by the Oδ1 atoms of both Asp25, with the catalytic water bound by the charged Oδ2 atoms in ligand-free HIV protease [42]. Binding of a substrate mimic induced an asymmetric conformation, shifting the proton to one of the Asp25. Earlier QM/MM simulations of aspartic peptidases focused on complexes of HIV protease with known or potential inhibitors from pharmaceutical research, such as nelfinavir, mozenavir, tipranavir, and the clinically most successful saquinavir [43,44]. In all cases, fine details of side-chain interactions were detected on the atomic level, which could improve the efficacy of the corresponding modified compound. In a comprehensive investigation of nine U.S. Food and Drug Administration (FDA) approved drugs, the causes for the reduced binding of most of them to the South African C subtype (C-SA) were revealed in atomic detail [45]. Some HIV protease inhibitors only gained academic interest, e.g., the metallacarborane complexes, exhibiting two cage-like C_2_B_9_H_11_ coordinated by a Co^2+^ center [46,47]. A step beyond was an NMR study combined with QM/MM supported modeling of more than dozen pentacycloundecane lactam-peptides and peptoids, which bind to the active site of HIV protease [48]. An investigation of two distinct starting conformations of HIV protease with the proton bound at Asp25B showed two pathways, one with proton transfer from the catalytic water to Asp25A, the other with a corresponding transfer to Asp25B [49]. The first pathway was even more favorable, when the amide hydrogen of the scissile bond interacted with the water O atom transiently, which reduced the free activation energy ∆G^‡^ to 69.0 kJ/mol compared to the experimental value of 66.5 kJ/mol. Previously, a more elaborate mechanism for HIV protease with three transition states was suggested, based on kinetic measurements with isotope labeling and ONIOM calculations [50]. However, simulation of a concerted one-step mechanism in HIV protease subtypes B and C-SA for the cleavage of peptides derived from the natural substrates Gag and Gag-Pol, resulted in excellent concordance with experimental values of the free activation energy barriers [51]. Using the so-called umbrella sampling QM/MM method, computed and experimental values came even closer [52]. Moreover, HIV protease ligand complexes of leukemia-related HTLV-1 protease were compared with respect to natural mutations [53].

A recent comparative study of HIV-1 protease and pepsin investigated the mechanism of the epoxide ring opening in an covalent inhibitor, employing the semi empirical self-consistent charge density functional tight binding (SCC-DFTB, DFTB3) quantum method in a hybrid QM/MM approach [54]. The calculations suggested a two-step mechanism for the epoxide ring opening and an unexpected oxyanion intermediate, which was stabilized by four co-catalytic water molecules in the protein active site, which had been identified in a previous study on HIV protease inhibitor bonds, using an electron localization function (ELF) based on DFT [55]. As the simulations resulted in covalent inhibitor complexes, which were known from structural data, it is very likely that this new oxyanion intermediate is part of the mechanism in aspartic proteases. By contrast, the comparison of substrate and inhibitor complexes of retropepsins and molecular orbital calculations revealed substrate assisted catalysis by an n→π* interaction with carbonyl O of the P1 residue as donor and the carbonyl C of the P1′ as acceptor, functioning as substitute of an oxyanion hole [56].

## 4. Mechanisms of Metalloproteases

**Figure 3:**
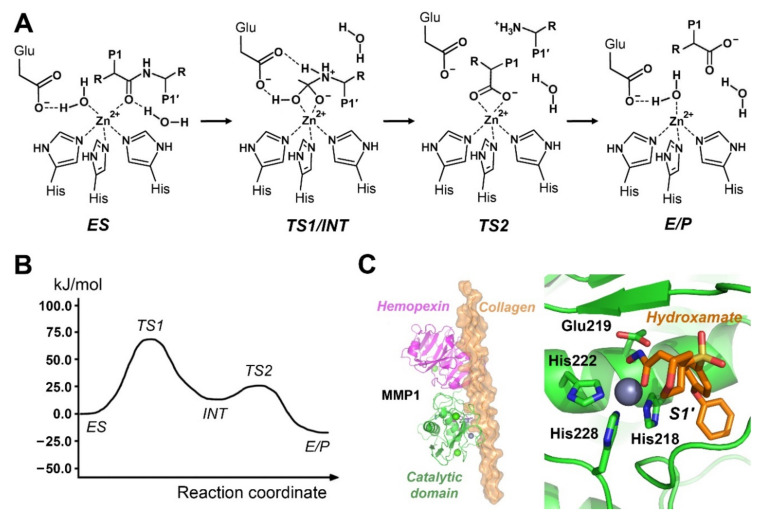
**(A)** Mechanism of metalloproteases. 1. Formation of a Michaelis complex (ES) and binding of the carbonyl O of the P1 residue to the catalytic Zn^2+^, which functions as oxyanion hole. 2. Nucleophilic attack by an activated water molecule, i.e., OH^−^ (TS1), formation of a tetrahedral intermediate and transfer of an H^+^ to the amide NH of the scissile bond (INT) 3. Cleavage of the scissile bond (TS2) and product release (E/P). Especially step 2 can be further subdivided into more steps. Structural examples with functional relevance are shown in Figure 3C. (**B**) The relatively simple free energy profile for MMP3 follows Pelmenshikov and Siegbahn (2002) [87]. (**C**) Left panel: MMP1 in complex with the natural substrate collagen (PDB 966C) corresponds to the ES complex. Right panel: MMP1 active site with a Zn^2+^ bound hydroxamate inhibitor (PDB 4AUO), which partially resembles the intermediate (INT).

Usually, researchers focus on mechanistic studies of the cancer-related human matrix metalloproteinases (MMPs). They possess a Zn^2+^ ion in catalytic center which is coordinated by three His residues, and a nearby Glu as activator of a catalytic water molecule. Other metalloprotease exhibit variations in these essential elements, however, the basic three-step proteolytic mechanism is very similar (Figure 3). An overview of metal catalyzed peptide bond hydrolysis by natural proteases and synthetic analogues is given by Zhang and coworkers [57]. The specificity of most MMPs is surprisingly similar, which makes it very difficult to design the much desired inhibitors that target individual MMPs, which are involved in cancer. Thus, the binding of inhibitors to various metalloproteases was investigated using QM/MM approaches since around the year 2000, e.g., for MMP1, MMP3, MMP9, carboxypeptidase A, and glutamate carboxypeptidase II [58,59,60,61,62,63,64,65,66,67]. A comparative study on MMP1-3, MMP8, MMP9, and MMP13 confirmed the good correlation of computed and experimental free binding energies of inhibitors, while a cross-docking matrix gave hints to rational design of more specific MMP inhibitors [68]. Unfortunately, MMP1-3, MMP7-9, and MMP12-14 exhibited nearly identical cleavage preferences for P3 to P3′ residues with little deviations in a proteomic specificity profiling: Pro-Ala-Xaa↓Leu-Val-Ala/Gly is sort of a collagen derived consensus sequence [69]. Another reason for the failure of MMP inhibitors as efficient drugs in clinical trials was the inhibition of several MMPs that have anti-tumor effects [70]. However, fine tuning of such inhibitors seems possible by exploiting the pronounced variability of the large S1′ pocket of most MMPs, which allows for excluding cross-reactivity with the so-called anti-targets, i.e., MMPs 3, 8, 9 and 14, due to their anti-tumor activity [71]. In the last decade, new MMP inhibitors and in vivo probes, namely specific ^99^Tc radiotracers, could be improved, such as in case of phosphinic compounds directed against MMP12, with a more than 200-fold selectivity compared to nine other MMPs [72,73]. Overall, the recent development for several MMPs is promising, as indicated by an increasing number of clinical trials [74].

As a basis for the reaction mechanism, the Zn^2+^ coordination sphere of MMP2 was chosen for QM calculations, resulting in the following plausible models with respect to the catalytic steps: octahedral coordination by His403, His407, His413, three H_2_O (one is bound to Glu404), bipyramidal with the three His and two H_2_O (Glu404), bipyramidal with an OH^−^ (Glu404) instead of an H_2_O and tetrahedral with the three His and one OH^−^ (Glu404) [75]. These four Zn^2+^ coordination variants are energetically deviating only by less than 10 kJ/mol. Essentially, a QM/MM study for MMP2 (gelatinase 1) assuming a two-step mechanism resulted in an activation energy barrier around 62 kJ/mol, very close to the measured values (Figure 3) [76]. Later, the same authors extended the reaction mechanism by using the formation of Michaelis complexes as starting point for the hydrolysis of collagen-like peptides to four transition states [77]. The simulated reaction of MMP2 with the substrate Ace-Gln-Gly↓Ile-Ala-Gly-Nme followed a similar mechanism with four transition states [78]. The same authors extended their work on MMP2 by repeating their calculations for the Glu116Asp mutant, with a slightly elevated ∆G^‡^ value of about + 4.0 kJ/mol [79]. In addition, inhibitor reaction mechanisms were computed for MMP2 and sulfoxide thiiranes, such as SB-3CT, and the oxirane analogs [80,81,82]. Considering thermodynamic differences between tautomeric forms, a QM based simulation of S1′ binding inhibitors could rationalize the Ki values in the lower nanomolar range by the dominating and favorable contribution of the free reaction enthalpy [83]. Supported by QM calculations a series of 20 novel tetrahydro-β-carboline inhibitors directed against the gelatinases MMP2 and MMP9 reached IC_50_ values in the pM range, whereas the inhibitory capacity showed mostly a reduced reactivity against MMPs 1, 3, 8, 12, 13, and 14 [84]. A related approach with virtual screening based on both pharmacophore modeling and molecular docking, identified four compounds from a library that inhibited MMP9 in the medium picomolar range [85]. By turning the natural MMP9 substrate regesepin-1 into double Cys variant, the now inhibitory compound bound the catalytic Zn^2+^ forming the zinc finger motif Cys_2_His_2_, accompanied by displacement of the third His ligand, which may open a route to more potent compounds [86].

Similar studies were performed for MMP1 with natural plant derived flavonol inhibitors [88]. Nevertheless, mechanistic studies continued, as for the catalytic domain of MMP3 (stromelysin 1), comprising the Zn^2+^ ligands His201, His205, His211, and the general base Glu202, in a simulation with a two-step reaction with two transition states [87]. Despite employing the very basic peptide mimic N-methyl acetamide, it yielded an activation energy barrier of 54.8 kJ/mol, in acceptable congruence with experimental data. As it is still debated, whether the nucleophilic water or the carboxylate of Glu202 of MMP3 forms the tetrahedral intermediate, a more recent study calculated both alternative reactions for the substrate Gly-Pro-Leu-Ala↓Thr-Cys-Val-Pro [89]. It turned out that a two-step reaction with an initial nucleophilic attack by the activated water molecule was more likely and energetically favorable. Recently, a novel QM/MM approach build promising inhibitory compounds from scratch, which are directed against membrane-bound and multiple myeloma-related MMP15 (MT2-MMP) [90].

The QM/MM simulated cleavage of a tripeptide by thermolysin followed the three-step mechanism, in which Glu143 deprotonates a water molecule prior to the nucleophilic attack, then the proton is transferred to the amide N atom, and the peptide bond breaks as in the two-step mechanism (Figure 3) [91]. Thereby, the calculated and measured free activation energies ∆G^‡^ differ to some extent with values of 61.9 and 54.0 kJ/mol, respectively. A mechanistic study using ONIOM QM/MM calculations was performed for carboxypeptidase A and its reaction with an inhibitor [92]. Supported by a peptide complex of the inactive mutant Glu424Ala, the simulated four-step mechanism of the prostate cancer-related glutamate carboxypeptidase II (GCPII) exhibited a 20% higher free activation barrier [93]. Insulin degrading enzyme is an ATP regulated protease, which cleaves the amyloid protein Aβ42 in a two-step mechanism, with calculated and measured ∆G^‡^ values in good agreement [94,95]. The catalytic mechanism of carboxypeptidase A for the hydrolysis of ester substrates was investigated with a hybrid QM/MM approach and high-level DFT [96]. In this special case, a nucleophilic attack by the catalytic Glu is an alternative to the so-called promoted-water pathway, which is the only productive one in the cleavage of peptide bonds.

## 5. Mechanisms of Cysteine Proteases

**Figure 4:**
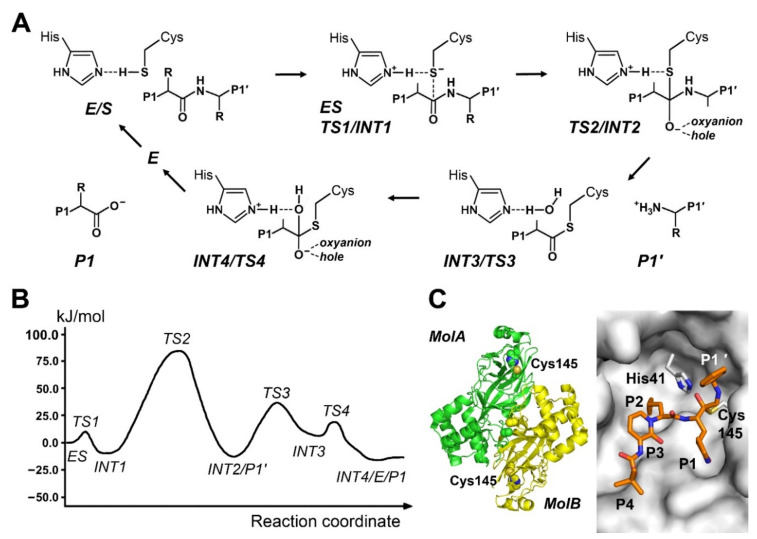
Mechanism of cysteine proteases. (**A**) As in the prototypic papain the catalytic residues of the dyad are a Cys and a His. Essentially two major reaction steps take place, namely the acylation and the deacylation, while several sub steps are involved, according to Wei et al. (2013) [99]. 1. Nucleophilic attack by the negatively charged Sγ atom on the carbonyl C of the P1 residue (TS1) and formation of tetrahedral intermediate (INT1). The oxyanion hole stabilizes the negative charge at the carbonyl O atom. 2. Upon protonation of the amide NH group the scissile bond breaks and the P1′ product leaves with a new N-terminus (TS2). 3. The acyl intermediate (INT2) is attacked by the nucleophilic catalytic water (TS3) and forms the second tetrahedral intermediate (INT3). 4. Release of the P1 product with a new carboxy terminus (TS4/INT4 and E/P1). (**B**) Free energy profile of the above described reaction. (**C**) The coronavirus SARS-Cov-2 main protease (M^Pro^) is depicted as free enzyme dimer (PDB 6Y2E) on the left and with a covalent ketoamide inhibitor (PDB 6Y2G) as TS2 analog on the right. Some residues of the protease were omitted for clarity.

In contrast to aspartic and metalloproteases, cysteine, serine, and threonine protease exhibit more transition states and intermediates, such as the relatively stable acyl intermediates. The latter ones facilitate transpeptidation reactions, in particular of cysteine proteases, with α-amino groups of peptides, which can be used for peptide ligation and related reactions [97]. In general, cysteine proteases possess specificity pockets in variations as the other protease classes. Papain from the plant *Carica papya* is the prototypic cysteine protease with a moderate trypsin-like preference for P1-Arg residues, exhibiting a specificity pattern for P2-P4′ Leu/Val-Arg/Lys↓Gln-Gln-Xaa-Asp [9,98]. Noteworthy, the oxyanion hole of the papain comprises the backbone NH group of the catalytic Cys25 and the side-chain CONH_2_ of Gln19 [12]. The first steps in the acylation reaction are not involving general base and acid catalysis by His159 of papain, which in turn is required for deacylation (Figure 4).

As early as 1990, a semi empirical QM/MM simulation of the acylation step alone demonstrated that the protonation of the scissile bond amide should take place before the nucleophilic attack of the Cys25 Sγ^−^, contrary to the above mentioned mechanism [100]. However, the a complete hydrolytic reaction was calculated in a hybrid quantum QM/MM study employing the minimal peptide substrate N-methyl-acetamide, which proceeded with a concerted protonation and nucleophile attack mechanism [101]. A slightly different approach favored the protonation of the substrate amide NH by His159 prior to the nucleophilic attack of Cys25 Sγ^−^ on the carbonyl C atom by comparing the free active site of papain with the N-methyl-acetamide complex [102]. Nevertheless, the free activation energy barrier remains nearly the same with calculated 83.7 kJ/mol, when chromogenic substrate N-acetyl-Phe-Gly-4-nitroanilide was hydrolyzed by separate steps of protonation and nucleophilic attack [99]. Both ∆G^‡^ values differ by about 11% from the lower experimental value of 74.9 kJ/mol. Employing N-methyl-acetamide as substrate, a more recent study supported the nearly concerted mechanism with the protonation starting right before the nucleophilic attack, with excellent concordance of computed and experimental ∆G^‡^ [103].

In total, 11 human cathepsin belong to the papain-like protease family and are mostly expressed in the acidic and reducing lysosomes [104]. In the non-prime side, their specificity for P1-Arg resembles papain, with an additional acceptance of P1-Gly. Apart from their diverse physiological roles, e.g., in the immune response, they gained interest due to their participation in diseases, such as rheumatism, inflammation, atherosclerosis, and various cancers. Since Cathepsin K (CatK) plays a role in these conditions, it is an interesting pharmacological target [105]. CatK exhibits the catalytic triad residues, which stabilizes the His residue during catalysis. Employing Acetyl-Leu-Arg-Phe-NMe as P2-P1-P1′ substrate, it turned out that the acylation step represents the highest free activation energy barrier energy with ∆G^‡^ = 122.6 kJ/mol, whereas the experimental ∆G^‡^ for the substrate (Abz-K-(LRF)-SKQ-EDDnp is about 57% of the calculated one [106]. Unexpectedly, the oxyanion hole, consisting of the Gln19 side-chain carboxyamide and the Cys25 backbone amide, only stabilized the thiolate nucleophile and not the carbonyl O of the scissile bond. Cathepsin B is expressed in lysosomes and involved in protection from apoptosis or diseases of the liver [105]. Regarding the ring opening of aziridine and epoxide inhibitors, such as E64c by CatB, a basic QM/MM approach showed that the proton transfer from His199 to the N and O atoms is energetically more favorable when mediated by a water molecule [107]. The water acts as efficient charge relay system, before the nucleophilic attack of the Cys29 Sγ^−^ results in formation of a covalent bond and irreversible inhibition. The ion pair of the catalytic dyad is stabilized by a H-bond network comprising Cys29 Sγ^−^, His199-H^+^, the oxyanion hole, i.e., the Gln23 carboxyamide and the Cys29 backbone amide, Ala200, Trp30, and up to four water molecules [108]. In the interaction of the intermediates with the oxyanion hole, the regio- and stereo-selective α-attack on the carboxylate C2 atom of the epoxide was more favorable, preferring the (S, S) configuration of the product [109]. Whereas QM/MM computed ruthenium arene complexes of CatB may be only of academic interest, a recent study combined crystallography with QM calculations, in order to compare the crystal interaction density and the electrostatic potential for CatB with the epoxide inhibitor loxistatin acid [110,111].

Another interesting pharmacological target is the chymotrypsin-like SARS-1 protease M^Pro^ (3CL^Pro^, clan PA), for which the proton transfer from Cys145 to His41 was systematically analyzed with a wide range of computational parameters [112]. It turned out that the DFT based QM/MM approach with the B3LYP functional was more robust than semiempirical ones. In addition, the reaction of SARS-1 M^Pro^ was simulated with a Michael acceptor, namely a trans-α,β unsaturated ethyl ester, as one-step mechanism [113]. In the current coronavirus pandemic 2021, the top priority target is the SARS-CoV-2 M^Pro^. A semiempirical QM/MM DFT calculation of the hydrolytic reaction of M^Pro^ dimer with the fluorogenic substrate Ac-Val-Lys-Leu-Gln-ACC exhibited four transition states, with excellent agreement of computed and experimental ∆G^‡^ values [114]. DFT based approaches were applied to a large set of pyridine N-oxide compounds as potential inhibitors of SARS-CoV-2 M^Pro^, peptidic Michael acceptor compounds, as well as to small molecule Schiff bases [115,116,117]. Moreover, SARS-2 M^Pro^ was virtually screened for inhibition by several hundred natural compounds [118]. Using DFT calculations treated by B3LYP/6-31G(d,p) flavonoid disaccharides, such as rutin, yielded the highest re-docking energy. A combined X-ray and QM/MM simulation for M^Pro^ with the sterol derived methide quinone celastrol showed a superoxide radical mechanism, resulting in a covalent bond to the catalytic Cys145 [119].

The related proteases falcipain and cruzain from *Plasmodium falciparum* and *Trypanosoma cruzi*, both infectious protozoans causing malaria and Chagas disease, respectively, reacted with epoxide inhibitors according to the above described mechanism [120,121]. Moreover, cruzain complex formation with covalent bond formation to nitrile inhibitors was similarly simulated, in good agreement with thermodynamic parameters ΔG, ΔH, and -TΔS, which were obtained from calorimetric measurements with the synthesized compounds [122]. A recent study of cruzain explained the irreversible and reversible binding of vinyl sulfone and nitrile inhibitors in terms of calculated thermodynamic parameters, which was supported by calorimetry data [123]. Similar results were obtained for the inhibition mechanism of the dipeptidyl nitroalkene Cbz-Phe-Ala-CβH=CαH-NO_2_ for the targets cruzain, rhodesain, and cathepsin L [124]. Rhodesain is another interesting drug target, as it is expressed in the parasite *Trypanosoma brucei rhodesiense*, which causes the African sleeping sickness. Since these three proteases share a chymotrypsin-like active site architecture, binding of the Michael system compound followed basically the same mechanism, starting with the nucleophilic attack of the catalytic Cys Sγ^−^ on the Cβ atom and followed by protonation of the Cα atom by the catalytic His. Remarkably, the free activation energy barriers for cruzain, rhodesain, and cathepsin L (64.9/85.4/188.7 kJ/mol) correlated well with the experimental inhibition constant K_i_ (0.44/0.49/11.0 nM), which was determined by the differences of the S1 and S2 specificity pockets.

Cysteine proteases from the clan CD comprise the caspases and multidomain paracaspases, which require dimerization for activation, whereas metacaspases and legumains are active as monomers [125]. Animal caspases and plant metacaspases are known for their intracellular activity in apoptosis and additional processes, such as inflammation [126]. Human caspases are highly interesting drug targets, due to their role in apoptosis and their unusual specificity for P1-Asp residues [127]. Caspases exhibit a different fold with respect to papain and require dimerization for full enzymatic activity. In case of caspase-3, the catalytic residues are Cys285 and His237, with the nearby hydrogen bond acceptor Thr177, while the backbone amide NH groups of Cys285 and Gly238 constitute the oxyanion hole. An QM/MM analysis of the nucleophilic attack of a water molecule on an acyl intermediate tripeptide, derived from the aldehyde inhibitor Ac-DEVD-CHO, yielded a free energy barrier of 79.5 kJ/mol, congruent with experimental data of 74.1 kJ/mol [128]. Legumains are caspase-related mammalian lysosomal and plant vacuolar cysteine proteases, which have become drug targets in cancer [129,130]. Contrary to mechanisms of papain-like proteases and caspases, human legumain requires a protonated catalytic Cys189 for the nucleophilic attack for the concerted formation of the thioester acyl intermediate [18]. The first intermediate is stabilized by a proper oxyanion hole, which is formed by the backbone NH groups of Gly149 and Als189, before a second nucleophilic attack of a catalytic water ensures the cleavage of the scissile bond. Depending on pH values < 6.0, legumain is a protease with a preference for substrates with P1-Asn and Asp, whereas above pH 6 transpeptidase or ligase activity is favored. 

Sortase A (SrtA) from *Staphylococcus aureus* exhibits a dual function: as transpeptidase it cleaves at the LPXTGG motif and ligates the remaining polypeptide-LPXTG acyl intermediate to peptidoglycans of the cell wall [131]. An ONIOM based simulation found that the catalytic His184 protonates the amide of the scissile bond prior to the nucleophilic attack of the Cys184 Sγ^−^, which is supported by Arg197 as equivalent to an oxyanion hole and Thr183 [132]. Computed and experimental ∆G^‡^ agree very well, whereby the mechanism comprises the two major steps of acylation an deacylation, while the acylation can occur in one concerted or two separate steps for the P2-Thr-*in* and Thr-*out* side-chain conformations [133]. The Thr-*in* conformation facilitated the separate protonation and nucleophilic attack mechanism with the lowest ∆G^‡^ value of 116.7 kJ/mol, which is still nearly 40% above the experimental value [133]. A recent study of tens of thousands of organic compounds for pharmacophoric screening was straightforward, in order to develop inhibitors of sortase A from the anthrax causing *Bacillus anthracis* (SrtA) [134]. Similar to sortases, the L,D-transpeptidases, such as LdtMt2 of *Mycobacterium tubercolosis,* catalyze peptide ligation, while they cleave β-lactam or peptidic bonds of antibiotics, conferring multidrug resistance [135]. The nucleophilic attack of Cys354 on the carbonyl C advances in a concerted manner with an energetically favorable 6-membered ring planar intermediate, including the OH moiety of a catalytic water [135].

## 6. Mechanisms of Serine Proteases

**Figure 5:**
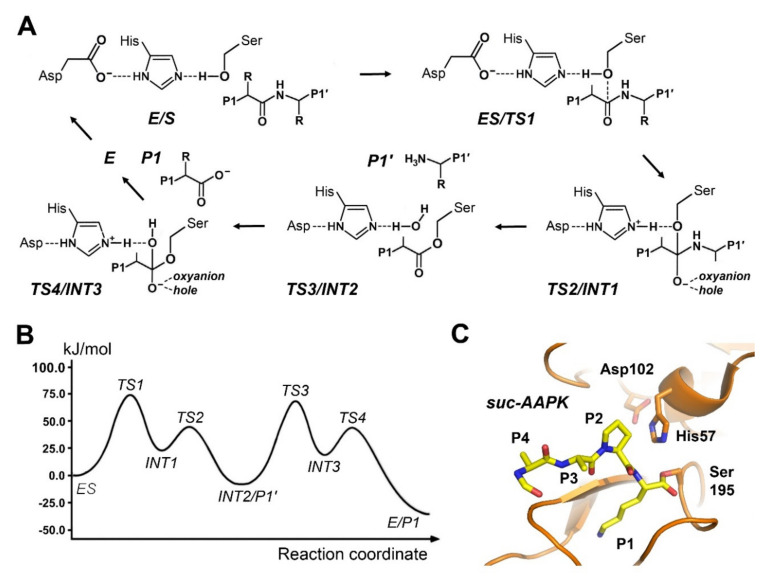
Mechanism of serine proteases. (**A**) The basic mechanism resembles the one of cysteine proteases. In a catalytic triad the acidic Asp is required to stabilize the positively charged His, which enhances the nucleophilicity of the Ser Oγ atom. 1. Formation of the Michaelis complex (ES). 2. Nucleophilic attack by the negatively polarized Oγ atom to the carbonyl C of the P1 residue (TS1), resulting in the tetrahedral intermediate with a negative charge at the carbonyl O (INT1), which is bound to oxyanion hole. 3. Protonation of the amide NH group breaks the scissile bond (TS2), with release of the of the P1′ product. 4. The acyl intermediate (INT2) is attacked by the catalytic water (TS3) and forms the second tetrahedral intermediate (INT3). 5. Release of the P1 product (TS4/E/P1) with a new C-terminus. (**B**) Free energy profile of the reaction in trypsin. (**C**) Trypsin in complex with a succinyl-Ala-Ala-Pro-Lys inhibitor (PDB 2AGG), which forms a true acyl intermediate, corresponding to INT2.

Serine proteases belong to the best characterized enzymes, with an encompassing understanding of their reaction mechanisms. The largest families, with respect to the protein fold, are the trypsin-like serine proteases, followed by the subtilase superfamily of the subtilisin-like peptidases and the α/β-hydrolases [136,137,138,139,140]. Besides some other serine protease families, they exhibit the classical catalytic triad of the Ser nucleophile, the general base and acid His, and a stabilizing Asp, which completes the charge relay system (Figure 5). The sequential order of the triad residues is different in these families, whereby additional residues may stabilize the triad during catalysis, such as the conserved Ser214 in trypsin-like proteases. In addition, there are serine proteases of smaller families, which exhibit a more diverse composition of their catalytic triads or dyads, as the example kumamolisin will demonstrate at the end of this chapter. The substrate specificity among these peptidases is often trypsin-like, chymotrypsin-like, and elastase-like, with a preference for P1-Arg/Lys, P1-Phe/Tyr, and P1-Val/Ala/Ser, respectively. However, there are many variations of the P1 specificity alone, such as in Granzyme B, which favors P1-Asp substrates. While the bacterial subtilisins are largely unspecific, the mammalian furins, and pro-protein convertases prefer basic P1 residues. By contrast, some α/β-hydrolases, such as dipeptidyl peptidase II, are specialized on P1-Pro substrates. Variations of the other specificity pockets are too multitudinous to be discussed here.

Ab initio QM/MM calculations for a porcine elastase complex with a natural heptapeptide substrate comprising the steps from the acyl intermediate and the nucleophilic water attack via the tetrahedral intermediate to deacylation, could largely disprove the previous idea of a His57 ring flip during catalysis [141]. Otherwise, the tetrahedral intermediate seemed stable in the picosecond range and was destabilized by a proton transfer from His57 to Asp102. Ishida and Kato found that the highest free activation energy barrier of about 75 kJ/mol belongs to the transition state before acyl intermediate formation, which lies right in the experimental range of ∆G^‡^ (63–84 kJ/mol) for various substrates [142]. Early kinetic studies in the year 1941 yielded already a ∆G^‡^ up to 67 kJ/mol [143]. This study favored a stepwise formation of the tetrahedral intermediate by an attack of the Ser195 Oγ nucleophile, followed by protonation of the scissile bond NH after the His57 side-chain had flipped. By contrast, these authors described the stabilizing effects of Asp102 on the transient His57-H^+^ and the surrounding active site residues [144]. A similar study on trypsin suggested a stepwise mechanism without His57 flip, whereas ∆G^‡^ was about 25% higher [145]. A thorough analysis of the His57 motion after the nucleophilic attack of Ser195 revealed that it requires only a subtle repositioning and no side-chain flip, in order to transfer the H^+^ to the NH group of the scissile bond, which elongates and starts to weaken before that substep [146]. Utilizing previous QM/MM results, Ishida calculated chemical shifts of the catalytic residues in trypsin for comparison with NMR data [147]. In addition, a simulated acylation step of trypsin upon cleavage of the cyclotide or cysteine-knot inhibitor MCTI-A was retarded, which was explained by the elevated free activation energy, caused by a hindered rotation of the NH group in scissile peptide bond [148]. A comparative analysis of 47 trypsin complexes with benzamidine derivatives reproduced free experimental binding energies ∆G very well, although it was limited to QM treatment of the ligands [149].

A structure based QM/MM study for about a dozen thrombin inhibitors, with experimental K_i_ values ranging from 0.56 to 4.60 nM, calculated K_i_ values, which overall correlated very well [150]. QM/MM predicted IC_50_ values for 64 pyridyl chromen-2-one derivatives as potential anticoagulants were compared with IC_50_ values derived from dose response curves of prothrombin time (PT) by Quick‘s method, resulting in a good linear correlation [151]. However, the data did not allow to determine which coagulation factors, in particular the serine proteases, might have been inhibited. Urokinase plasminogen activator (uPA) plays important roles in fibrinolysis and cancer processes and, thus, is an attractive pharmacological target. In total, five inhibitors carrying guanidino groups in the P1-residue displayed experimental K_i_ values from 180 to 0.41 µM, while QM/MM calculated K_i_ values correlated altogether very well [152]. The varying inhibitory potency of seven peptidyl α-ketoheterocyclic compounds for human neutrophil elastase (HNE) was explained by a range from covalent bond formation to no reaction at all with the Ser195 nucleophile [153].

Viral proteases are interesting targets for mechanistic and inhibitory simulation. For example the NS3 protease of the RNA hepatitis C virus (HCV) is a very short trypsin-like protease of 180 residues length, which requires the NS4A cofactor (54 aa). Although the mechanistic steps for substrate cleavage by the NS3 protease do not differ significantly from the previously described ones, the calculated ∆G^‡^ was about double the experimentally derived 78 kJ/mol, while preliminary simulations focused on the interaction with the NS5A/5B Substrate [154,155]. Computed free activation energies for other viral polyprotein substrates of NS3/NS4A, such as NS5A/5B, NS4B/5A, and NS4A/4B, did not abolish the discrepancy to the experimental data [156]. Intriguingly, further exploration of this system employed SCC-DFTB for the QM part, yielding a significantly lower ∆G^‡^ value, which differed only by 12% from the experimental one [157]. QM/MM computation of the HCV NS3/4A protease mutants R155K and D168A explained the increased resistance to the sulfonamide inhibitor asunaprevir by the loss of a hydrogen bond network [158]. Similarly, the reaction mechanism of the HCIV-related NS3 protease of the Dengue virus with its cofactor NS2B exhibited a stepwise nucleophilic attack and protonation at the scissile bond during the acylation process, whereby the energy barrier ∆G^‡^ of 100.8 kJ/mol seemed to be halfway in the range of the above described experimental data [159]. Moreover, the Zika virus (ZIKV) protease NS3 complex with the peptide Thr-Gly-Lys-Arg↓Ser, which is located at the C-terminus of the NS3 cofactor NS2B, follows the standard mechanism with a concerted nucleophilic attack of Ser135 and proton transfer to His51, yielding acceptable calculated ∆G^‡^ values [160]. The acyl-KR-aldehyde inhibitor of ZIKV NS3/NS2B protease forms a relatively stable covalent hemiacetal adduct and showed with five different QM/MM methods a consistent reaction mechanism and similar energetics [161].

Apparently, the reaction mechanism of furin differs from the trypsin-like proteases, as cleavage of the H5N1 avian influenza virus hemagglutinin involves a single transition state in a concerted reaction, with the H^+^ transfer from Ser368 to His194 together with the nucleophilic attack of the Ser368 Oγ on the carbonyl C of the scissile bond [162]. A different underlying mechanism proceeds in the transesterification of L-phenylalanine-N-acetyl-ethyl-ester, which is attacked by the Ser221 Oγ nucleophile of subtilisin Carlsberg with a concerted proton transfer to His64 [163]. The medium was hexane as apolar solvent (66%) mixed with water (34%), resulting in nearly equal calculated and measured ∆G^‡^ values. A very special case was the complex formation between subtilisin BPN′ and the chymotrypsin inhibitor 2 (CI2), followed by the proteolytic cleavage of CI2, whereby five transition states and several intermediates had to be considered [164]. As the acylation is reversible and access of hydrolytic water molecules is blocked at the active site, the final cleavage of CI2 is very slow, while the calculated and experimental rate constants of four mutants correlated very well.

Kumamolisin from the sedolisin protease family is an unusual acidic serine protease from the thermophile and acidophile *Bacillus novosp. MN-32*, which possesses a catalytic triad consisting of Ser278, Glu78, Asp164, with an atypical oxyanion hole formed by the Ser278 backbone NH group and the Asp164 Oδ1 atom [165]. It is related to subtilisins and shares about 13% identical residues with the subtislisin Carlsberg variant, whereby an optimal P4 to P4′ substrate might be Val-Glu-Ala-Leu↓Tyr-Leu-Val-Lys according to the MEROPS database [9]. In contrast to the crystallographic study, the backbone amide of Glu78 was proposed as part of the oxyanion hole during substrate-assisted catalysis of kumamolisin [166]. A more detailed simulation of the catalytic mechanism identified Asp164 as the general acid and base, which protonates the carbonyl O of the scissile bond in the second and rate-limiting substep of the acyl intermediate formation, after the nucleophilic attack of the Ser278 Oγ atom [167]. Glu78 was suggested to protonate the NH group of the scissile bond and activate the water nucleophile, while Asp164 again protonates and deprotonates the carbonyl O atom [168]. In addition, pro-kumamolisin, the zymogen form, adopts already an active conformation, which cleaves its own prodomain in an autocatalytic reaction that resembles the mechanism and energetics of the normal substrate cleavage [169].

## 7. Mechanisms of Threonine Proteases

Although only a handful threonine proteases are known, such as the proteasome and its bacterial counterpart HslV, they are ubiquitous, since the majority of living cells possesses them in hundreds of copies [170]. In eukaryotes, proteasomes degrade polyubiquitinilated proteins, thereby contributing to cell cycle control, protein homeostasis, inflammatory, and apoptotic processes, only matched by lysosomal protein degradation [171]. Many regulatory proteins interact with the 20S core particle, especially the ATPase complexes that associate to the 26S proteasome [172]. These AAA+-ATPases unfold the ubiquitinylated substrates and thread the polypeptide chains into the catalytic chamber of the 20S core for multiple proteolytic cleavages. The core is a barrel shaped assembly, which consists of four rings with seven subunits in the stoichiometry α_7_β_7_β_7_α_7_, while catalytically active Thr1 residues are only present in the subunits β1, β2, and β5. Subunit β1 exhibits a caspase-like specificity, preferring P1-Asp and Glu, β2 cleaves in a trypsin-like manner after basic P1 residues, and β5 is more chymotrypsin- or elastase-like, accepting hydrophobic residues from Ala, Leu, to Tyr [173]. Moreover, immunoproteasomes and thymoproteasomes with altered specificity generate antigens for presentation at cellular surface in major histocompatibility complexes class I (MHC-I). Based on the central roles as cytosolic protein degradation machinery and immunological control system, proteasomes are favored targets for inhibitory pharmaceutical compounds, which can be improved by understanding the reaction mechanisms on the atomic level.

**Figure 6:**
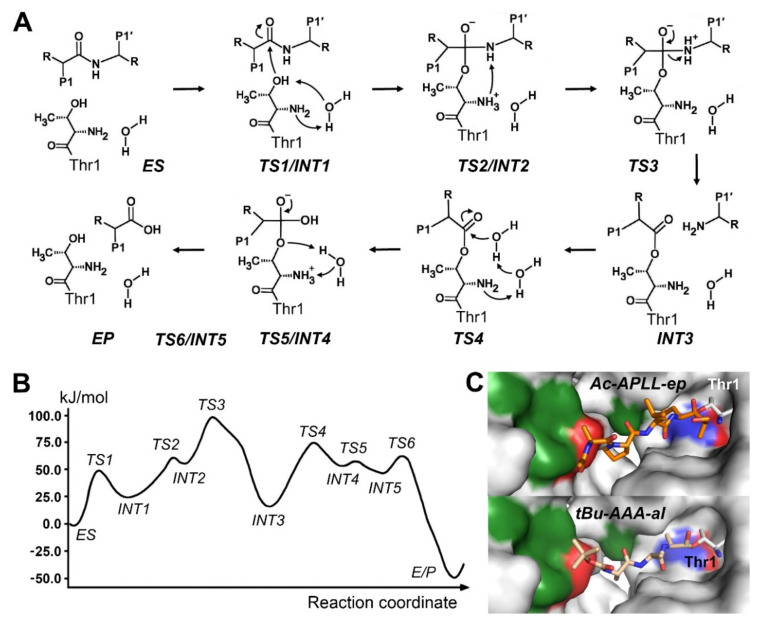
Mechanistic details of threonine protease catalysis. (**A**) The six step mechanism according to Wei et al., 2013 [175]. (**B**) Free energy profile of the six-step reaction. (**C**) Inhibitor structures of the yeast 20S proteasome. Upper panel: The epoxide Ac-Ala-Pro-Leu-Leu-ep covalently bound to the Thr1 Oγ atom of the β5 subunit corresponds to the tetrahedral intermediate INT2. The αN-C bond is not shown for clarity in the upper panel (PDB 4QBY). Lower panel: The tBu-Ala-Ala-Ala-aldehyde bound to Thr1 represents the acyl intermediate INT3 (PDB 4Y8L).

To date, two major studies were attempted to explore the proteolytic mechanism with first principles QM/MM free energy calculations. A forerunner of the first study was the investigation of the binding mechanism of the epoxide inhibitor epoxomicin to the active site, formed by a catalytically active β5 and inactive, but specificity pocket shaping β6 subunit of the yeast proteasome [174]. The same setting was employed in calculating the reaction mechanism for cleavage of the fluorogenic substrate succinyl-Leu-Leu-Val-Tyr-AMC, which comprises the following six steps: 1. H_2_O assisted H^+^ transfer to the Thr1-Nα, which activates the Thr1-Oγ; 2. nucleophilic attack of the Thr1-Oγ on the carbonyl C of the Tyr-AMC bond; 3. elimination of the amine product AMC by formation of the acyl Inter mediate; 4. nucleophilic attack of H_2_O on the carbonyl C of Tyr with H^+^ transfer to Thr1-Nα; 5. elimination of the suc-Leu-Leu-Val-Tyr-CO_2_^−^ product; 6. direct H^+^ transfer from the Thr1-Nα to Thr1-Oγ (Figure 6A) [175]. An alternative reaction mechanism was suggested for steps 3 and 4 by H_2_O assisted H^+^ transfer. Nevertheless, the favored six step reaction has a free energy barrier of 76.1 kJ/mol, which comes close to the experimental value of 78.9 kJ/mol (Figure 6B).

A recent publication was based on exactly the same system with suc-LLVY-AMC as substrate, whereas an empirical valence bond approach for the protein electrostatics was implemented [176]. Thus, Lys33 Nε as H^+^ acceptor and activator of the Thr1-Oγ and as donor of H^+^ to the Thr1-Nα appears to be more significant in a general base and acid scheme (Figure 6A). Thr1-Nα acts as second general base and acid, which transfers the H^+^ to the amide N of the scissile bond, resulting in a reaction with nine major steps. In contrast to the previous studies, the free energy calculation was coupled, taking into account the critical electrostatic interactions of the MM region on the QM treated atoms. The group of Saha found that the nucleophilic attack determines the reaction rate by the acylation and the deacylation free energy barriers ∆G^‡^, which were 60.7 and 87.9 kJ/mol. The value derived from an experimental k_cat_ with an archaean 20S proteasome was 77.8 kJ/mol, which is at least in the same range, although the turnover of small fluorogenic substrates and full length proteins differs significantly [177]. Addition of a proteasome activating ATPase (PAN) to the 20S samples enhanced the turnover of proteins about threefold.

## 8. Conclusions

In the last decades, many research topics in the area of molecular biology have progressed and improved by leaps and bounds. Due to the novel technological advances computational biology can provide fundamental insights into the underlying catalytic principles, which are difficult to elucidate or cannot be studied by experimental methods in an appropriate manner. The Nobel Prize in Chemistry in 2013 was awarded to Martin Karplus, Michael Levitt, and Arieh Washel in the field of computational biology, for the development of multi-scale models for complex chemical systems (QM/MM methods), indicating the importance of computer based simulations for biological and biomolecular processes. 

Combined quantum mechanics and molecular mechanics (QM/MM) methods are an effective approach to model the reaction mechanisms of enzyme-catalyzed reactions. This technique allows a detailed simulation of enzymatic reactions by coupling high level quantum chemical calculations on the active site with molecular mechanics treatment of the rest of the protein and the solvent. Different reaction mechanisms can be calculated, compared and analyzed. Overall, the mechanistic QM/MM hybrid studies concord astonishingly well in terms of calculated free energies, enthalpies, and entropies with corresponding parameters, which were either derived from kinetic, calorimetric, or isotope exchange measurements (Table 2). Usually, these simulations have a strong structural background with coordinates from the Protein Data Bank and in some cases they are supported by further experimental data from mutational and inhibitor binding investigations. 

Based on methodological progress and increasing computational power, recent simulations could reveal many fine details of proteolytic mechanisms, e.g., for papain, whereby the calculated and measured activation energies ∆G^‡^ are virtually equal [103]. Apparently, neither the separate substeps of the nucleophilic attack and protonation by the catalytic dyad of Cys and His nor the concerted mechanism of previous studies were fully correct. The refined model mechanism rather starts with the protonation, but the nucleophilic attack begins still during the protonation. Thus, a more complete picture represents sort of a compromise on a higher level between the initial, rather than opposing views.

Future X-ray crystallography and NMR iterative model building and refinement may benefit from QM/MM calculations, and result in more precise final biomolecular models [178]. In addition, corresponding in silico mutagenesis studies may help to eliminate protein mutants that have no or little effect on functional parameters, whereas mutants with more impact on kinetic and thermodynamic behavior can be purified and experimentally investigated. First steps in this direction were the theoretical mutational studies of cytochrome P450 with a selenocysteine replacing a cysteine and simulations of the L-asparagine V27T mutant, which should have a significantly lower glutaminase activity [179,180].

An increasing number of QM/MM calculations investigates the interaction of inhibitors with protease targets, which are of medical and pharmacological interest, exemplified by ranking calpain-1 and -2 or rhodesain inhibitors according to the computed binding capacity [181,182]. This kind of research may greatly benefit from the hybrid QM/MM approaches, which can provide valuable information on the relevant transition states and intermediates of the proteolytic reaction and may guide the design of more stable inhibitors serving as potent pharmaceutical drugs for both substrates and inhibitory compounds [183]. Probably, more detailed information can be obtained, such as the prediction of k_on_ and k_off_ rates, for both substrates and inhibitory compounds, which could be related to experimental data, such as K_M_. Moreover, such computational developments can contribute significantly to expand the applications of several enzymes in industry. Combined QM/MM methods will elucidate key information concerning the enzyme mechanism and can even pave the way for successful rational enzyme re-design.

## Figures and Tables

**Table 1:** Thermodynamic parameters of proteases at 298.15 K in kJ/mol. The models were BACE2 (Asp), MMP9 (Metallo), cruzain (Cys), hepatitis C virus NS3/NS4A (Ser), 26S proteasome (Thr).

Protease	PDB ID	ΔG^‡^	ΔH^‡^	ΔS^‡^
Uncatalyzed reaction		183.7	172.4	−38.1
Aspartic protease	2EWY	150.6	132.2	−61.1
Metalloprotease (Zn^2+^)	1L6J	128.4	114.2	−48.1
Cysteine protease	1AIM	54.8	46.4	−28.5
Serine protease	1DXP	108.8	96.7	−40.6
Threonine protease	6MSB	56.5	52.3	−13.4

**Table 2:** Calculated free energy values for protease reaction mechanisms using QM/MM hybrid simulations. PDB codes, level of theory of QM/MM method with either the software package or the force fields, and calculated and experimental ∆G^‡^ in kJ/mol are shown. ^1^ subtype B; ^2^ concerted acylation; ^3^ separate protonation and nucleophilic attack during acylation; ^4^ concerted mechanism with initial protonation; ^5^ various QM approaches, the substrates of the calculations and experiments differ significantly; ^6^ according to the k_cat_ for the substrate Ac-D-Tyr-Tic-Thr-Asn-ACC, whereby Tic is an unnatural imino acid [127]; ^7^ combined with basis set 6-31G(d) ^8^ average value of various substrates; ^9^ transesterification in hexane–water (2:1) ^10^ various QM submethods were compared.

Protease	PDB	Method QM [basis Set)]/MM (Package)	ΔG^‡^_calc_	ΔG^‡^_exp_	Citation
HIV protease	4HPV	ONIOM [B3LYP, 6-311+g(2d,2p)]/AMBER10	69.0	66.5	[49]
HIV protease B ^1^	23PB	ONIOM [B3LYP/6-31++G(d,p)]/AMBER10	63.6	65.7	[51]
Renin (mouse)	1SMR	ONIOM [B3LYP/6-31G(d)]/AMBER10	99.2	82.0	[33]
MMP2	1CK7	DFT [B3LYP, 6-31G*,LACVP*]/AMBER9	61.9	64.4	[76]
MMP2	1CK7	DFT [B3LYP/LACVP*]AMBER9	71.5	67.8	[77]
MMP2	1QIB	DFT [PBE0-D3/B3LYP, 6-31G**]]/CHARMM	69.5	66.9	[78]
MMP3	1B8Y	ONIOM [B3LYP, 6-311+G-(1d,1p)]	54.8	65.9	[87]
MMP3	1M1W	DFT [SIESTA/BLYP3, 6-31G⁄]/AMBER10	61.9	65.9	[89]
Thermolysin	1LNF	DFT [[B3LYP, 6-311++G(d,p)]/CHARMM	61.9	54.0	[91]
GCPII	2C6C	DFT [B3LYP, def2-TZVP]/AMBER8	92.0	79.5	[93]
IDE	2WK3	DFT [SCC-DFTB]/AMBER12	62.8	62.0	[95]
Papain ^2^	9PAP	QM-AM1 [B3LYP, 3-21G*]/AMBER4	84.1	74.9	[101]
Papain ^3^	1KHP	DFT [B3LYP, 6-31++G**]/AMBER8	83.7	74.9	[99]
Papain ^4^	1PPN	ONIOM [B3LYP, 6-31++G(d,p)]/AMBER12	75.7	74.9	[103]
Cathepsin K ^5^	1AYU	QM [B3LYP, 6-311+G(d,p)]/CHARMM22	122.6	69.5	[106]
SARS-CoV-2 M^Pro^	6LU7	QM-AM1 [M06-2X, 6-31+G(d,p)]/AMBER	83.3	81.2	[114]
Caspase-3	1PAU	DFT [BLYP]/GROMOS96	79.5	74.1	[128]
Legumain	4AW9	DFT [B3LYP, Ahlrichs-pVDZ]/AMBER99	68.6	78.3 ^6^	[18]
Sortase A ^7^	2KID	ONIOM [B3LYP, 6-311+G(2d,p]/AMBER03	81.2	83.7	[132]
LdtMt2	3VYP	ONIOM [B3LYP/6-31+G(d)]/AMBER96	102.7	102.1	[135]
Trypsin	1MCT	MP2 [(aug)-cc-pVDZ/ HF, 6-31+G**]/AMBER	74.5	72.3 ^8^	[142]
Furin	1HA0	AM1/PM3 [B3LYP/6-31+G*]/CHARMM22	67.8	73.2	[162]
Subtilisin C ^9^	1VSB	MP2 [BMK, 6-311+G**]/CHARMM27	65.7	67.8	[163]
NS3/NS4A-HCIV	1DXP	QM [AM1-SE]/CHARMM22	149.1	78.1	[154]
NS3/NS4A-HCIV	1DXP	QM [SCC-DFTB]/CHARMM22	87.9	78.2	[157]
NS3/NS2B-ZIKV ^10^	5GJ4	QM [BH and HLYP-D3, 6-311++G(d,p)]/AMBERFF14SB	68.2	76.1	[160]
β5/β6 proteasome	1G65	QM-HF [B3LYP, 6-31++G**]/AMBER8	76.1	78.9	[175]

## Data Availability

No new data were created or analyzed in this study. Data sharing is not applicable to this review article.
